# Socioeconomic factors, medication subsidisation and the use of preventative cardiovascular disease medications in Australia.

**DOI:** 10.23889/ijpds.v7i3.1982

**Published:** 2022-08-25

**Authors:** Ellie Paige, Emily Banks, Jason Agostino, David Brieger, Karen Page, Grace Joshy, Eden Barrett, Rosemary Korda

**Affiliations:** 1 Australian National University; 2 Concord Clinical School, The University of Sydney; 3 Deakin University, Melbourne

## Objectives

Cardiovascular disease (CVD) events are highly preventable through appropriate treatment and disproportionally affect socioeconomically disadvantaged individuals. This study quantified the relationship of socioeconomic factors to dispensing and persistent use of lipid- and blood pressure-lowering medication following hospital admission for a major CVD event (myocardial infarction, ischaemic stroke/transient ischaemic attack).

## Approach

Data from 8,285 people with major CVD events aged ≥45 years from the Australian 45 and Up Study with linked medication dispensing data were used. Modified Poisson regression was used to estimate relative risks (RRs) for combined lipid- and blood pressure-lowering dispensing at three-months following hospital discharge and for 12-month persistent use, in relation to education, income, and level of medication subsidisation.

## Results

Overall, 56% were dispensed guideline-recommended medications at three months and 37% persistently used them across 12 months. After adjusting for demographic factors, type of CVD and history of CVD hospitalisation, RRs for lowest (no educational qualifications) compared to highest education level (university degree) were 1.14 (95% CI: 1.06, 1.22) for medication dispensing and 1.15 (1.02, 1.29) for persistent medication use; 1.14 (1.06, 1.22) and 1.17 (1.04, 1.32) respectively for lowest (<$20,000) versus highest (≥$70,000) household pre-tax income; and 1.25 (1.17, 1.33) and 1.28 (1.15, 1.43) respectively for those receiving highest versus lowest subsidisation. There was little to no evidence of a relationship of income and education to medication use after adjustment for medication subsidisation.

## Conclusion

While preventive medication use is sub-optimal, subsidisation is substantially associated with increased use and accounts for most of the relationship with socioeconomic position, suggesting medication subsidy schemes are working in the intended direction.

